# Increased 14-3-3β and γ protein isoform expressions in parasitic eosinophilic meningitis caused by *Angiostrongylus cantonensis* infection in mice

**DOI:** 10.1371/journal.pone.0213244

**Published:** 2019-03-07

**Authors:** Hung-Chin Tsai, Yu-Hsin Chen, Chuan-Min Yen, Susan Shin-Jung Lee, Yao-Shen Chen

**Affiliations:** 1 Section of Infectious Diseases, Department of Medicine, Kaohsiung Veterans General Hospital, Kaohsiung, Taiwan and National Yang-Ming University, Taipei, Taiwan, R.O.C.; 2 Department of Parasitology and Graduate Institute of Medicine, Kaohsiung Medical University, Kaohsiung, Taiwan, R.O.C.; University of Toledo Health Science Campus, UNITED STATES

## Abstract

The 14-3-3 proteins are cerebrospinal fluid (CSF) markers of neuronal damage during infectious meningitis and Creutzfeldt-Jakob disease. Little is known about dynamic changes in the individual isoforms in response to parasitic eosinophilic meningitis. The purposes of this study were to determine the 14-3-3 protein isoform patterns, examine the kinetics and correlate the severity of blood brain barrier (BBB) damage with the expressions of these markers in mice with eosinophilic meningitis.

Mice were orally infected with 50 *A*. *cantonensis* L3 via an oro-gastric tube and sacrificed every week for 3 consecutive weeks after infection. The Evans blue method and BBB junctional protein expressions were used to measure changes in the BBB. Hematoxylin and eosin staining was used to analyze pathological changes in the mice brains following 1–3 weeks of infection with *A*. *cantonensis*. The levels of 14-3-3 protein isoforms in serum/CSF and brain homogenates were analyzed by Western blot, and immunohistochemistry (IHC) was used to explore the different isoform distributions of 14-3-3 proteins and changes in BBB junctional proteins in the mice brain meninges. Dexamethasone was injected intraperitoneally from the seventh day post infection (dpi) until the end of the study (21 dpi) to study the changes in BBB junctional proteins. The amounts of Evans blue, tight junction and 14-3-3 protein isoforms in the different groups of mice were compared using the nonparametric Kruskal-Wallis test.

There were significant increases in 14-3-3 protein isoforms β and γ in the CSF in the second and third weeks after infection compared to the controls and first week of infection, which were correlated with the severity of BBB damage in brain histology, and Evans blue extravasation. Using IHC to assess the distribution of 14-3-3 protein isoforms and changes in BBB junctional proteins in the mice brain meninges, the expressions of isoforms β, γ, ε, and θ and junctional proteins occludin and claudin-5 in the brain meninges increased over a 3-week period after infection compared to the controls and 1 week after infection. The administration of dexamethasone decreased the expressions of BBB junctional proteins occludin and claudin-5 in the mice brain meninges.

Our findings support that 14-3-3 proteins β and γ can potentially be used as a CSF marker of neuronal damage in parasitic eosinophilic meningitis caused by *A*. *cantonensis*.

## Introduction

*Angiostrongylus cantonensis*, also known as rat lungworm, is the main etiology of human eosinophilic meningitis or meningoencephalitis in southeast Asia and the Pacific region [1–5]. Humans are infected with *A*. *cantonensis* by ingesting freshwater and terrestrial snails and slugs [5–8]. The major intermediate hosts for *A*. *cantonensis* in Taiwan are the African giant snail (*Achutina fulica*) and golden apple snail (*Ampullarium canaliculatus*) [4, 7–8]. When infection occurs in non-permissive hosts such as humans and mice, the development of the parasites will stop at the young-adult worm stage, causing brain damage and inducing eosinophilia in the blood and cerebral spinal fluid (CSF) [8–10]. In addition, brain apoptosis [11], the presence of 14-3-3 β protein [12] and dysfunction of the blood brain barrier (BBB) with CSF eosinophilia have been reported in mice and humans infected with *A*. *cantonensis* [8–10, 13].

Seven isoforms of 14-3-3 protein have been identified in the brain, and they have been reported to be involved in many cell functions including cell cycle and transcriptional control and intracellular trafficking, as described in the previous study [14]. High levels of the isoforms of 14-3-3 have been reported in brain tissue, especially in Purkinje cells in the cerebellum (the η isoform) [15]. In addition, high levels of the β and γ isoforms have been reported to be more brain specific [16–18]. In the normal murine brain, the β, γ, η and ζ isoforms are distributed mainly in neuronal cell bodies, and often in particular anatomical nuclei. Individual isoforms have been shown to have subtle differences in location. For example, the τ isoform is found only in the hippocampus and medulla, whereas the ε isoform is found throughout the grey matter of the central nervous system (CNS) as described in the previous study [19]. The presence of 14-3-3 proteins in the CSF has been reported in patients with Creutzfeldt-Jakob disease (CJD) [20], and it is thought to result from neuronal disruption and leakage of brain proteins into the CSF [21]. These proteins have also been found in the CSF of patients with various neurological diseases, including meningitis [22, 23]. In addition, CSF total leukocyte and eosinophil counts were positively correlated with BBB dysfunction in a mice study of eosinophilic meningitis [13]. Another previous study also demonstrated that the 14-3-3 proteins could be considered a neuropathological marker of neuronal damage in bacterial meningitis [23]. Although the lack of specificity compared to CSF eosinophilia clearly implies substantial limitations in the use of 14-3-3 proteins as a specific disease marker, their value as an indicator of neurological damage could be used to monitor the evolution of diseases such as eosinophilic meningitis.

In this study, we examined dynamic changes in the expressions of various 14-3-3 protein isoforms in a mice model with eosinophilic meningitis, and correlated them with the Evans blue test, tight junctional protein changes and brain immunohistochemistry (IHC) findings.

## Materials and methods

### Ethics statement

The Animal Committee of Kaohsiung Veterans General Hospital approved the study protocol. All animal studies were conducted in strict accordance with the recommendations of the Animal Protection Act of Taiwan.

### Infection of Balb/C mice

Forty Balb/C mice (20 male and 20 female) aged 6–7 weeks were purchased from the National Laboratory Animal Breeding Research Centre. They were housed in groups of 3–5 in cages under a 12 h light/dark cycle and maintained in an air-conditioned animal facility (25±2°C and 50±10% relative humidity) with unrestricted access to sterilized food and water. Third-stage larvae of *A*. *cantonensis* were harvested using a method previously described [13]. Briefly, the mice were orally infected with 50 *A*. *cantonensis* L3 via an orogastric tube after slight ketamine anesthesia. Five mice were sacrificed every week for 3 consecutive weeks after infection until the end of the study. Dexamethasone at a dose of 500 μg/kg/day was injected intraperitoneally from the seventh day post infection (dpi) until the end of the study (21 dpi). No deaths occurred apart from the planned euthanasia. Infection was confirmed in all of the mice by the presence of larvae in CSF sediments.

### Collection of serum and CSF specimens

Blood samples from the mice were collected by heart puncture under ketamine anesthesia. The brains were removed and washed with 50 μL 0.15 M phosphate buffered saline (PBS), and the cerebral ventricles and cranial cavities were washed with 350 μL PBS. The supernatant was stored at -70°C until further analysis. The detailed procedure was described in our previous study [12].

### Measurement of permeability of the blood-brain barrier using the Evans blue method

The detailed study protocol was described in a previous study [24]. Briefly, a volume of 200 μL of 2% (w/v) solution of Evans blue (Sigma, St. Louis, MO, USA) in PBS was administered intraperitoneally. After 2 hours circulation, the mice brains were removed after anesthesia with ketamine and ground with 1.0 mL PBS in a glass-tissue grinder with a Teflon pestle. The extract was then centrifuged at 18,000 *g* (Hermle, Z326K, Germany) for 10 minutes at room temperature. The optical density of the supernatant was read at 595 nm using a colorimeter (Thermo Scientific Multiskan FC, USA) [24].

### Dynamic changes of 14-3-3 protein isoforms in CSF/serum/brain homogenates and expressions of tight junctional proteins occludin and claudin-5 in CSF by Western blot analysis

Whole brains were homogenized in lysis buffer (10 mM HEPES at pH 7.9, 10 mM KCl, 1.5 mM MgCl2, and a mixture of protease inhibitors, including phenylmethylsulfonyl fluoride, aprotinin, leupeptin, and pepstatin A) and centrifuged at 12,000 g for 15 minutes at 4°C. In brief, the supernatants were diluted 1:1 (vol/vol) with sample buffer (125 mM Tris-HCl, 4% SDS, 0.05% bromophenol blue, 20% glycerol, and 5% b-mercaptoethanol) and heated to 90°C for 10 minutes. Duplicate 15-μL samples of CSF and serum were used. They were then separated by electrophoresis on 10% Bis-Tris gels in SDS buffer. After transfer, PVDF membranes were probed at room temperature with mouse monoclonal IgG for pan 14-3-3, rabbit polyclonal IgG antibody for 14-3-3 β, ε, γ, η, ζ, τ, θ and σ (Santa Cruz Biotech, Santa Cruz, CA), rabbit polyclonal antibody for occludin (Abcam, Cambridge, UK) and rabbit polyclonal antibody for claudin-5 (Santa Cruz Biotech, Santa Cruz, CA), followed by goat anti-rabbit HRP-conjugated antibody (Santa Cruz Biotech, Santa Cruz, CA). The blots were developed using an enhanced chemiluminescent system (Amersham; GE Healthcare, UK). Densitometric values for each sample were obtained using a computer-assisted laser scanner (GS-710 Calibrated Imaging Densitometry; BioRad, BioSurplus, USA) after correcting for the background. The total amount of 14-3-3 protein as quantified in each diluted and undiluted sample was expressed in arbitrary units. Control specimens included samples obtained from Balb/C mice without infection.

### Histopathological examination of brain tissue after 1, 2, and 3 weeks infection with *A*. *cantonensis*

One control animal and one infected animal were sacrificed after 1, 2 and 3 weeks, respectively. The brains were removed from the cranial cavity and fixed in 10% formalin, and then embedded in paraffin. Each brain was cut into five portions including the anterior cerebrum, lateral ventricles, third ventricle and hippocampus, posterior cerebrum, and fourth ventricle and cerebellum. Coronal slices (4–5 μm thick) were made and stained with hematoxylin and eosin. The stained slices were examined under a light microscope.

### IHC for 14-3-3 and tight junctional proteins

To identify the cellular localization of 14-3-3 and tight junctional proteins occludin and claudin-5 in the brain meninges, coronal sections of brain tissue were immunohistochemically stained with a polyclonal antibody directed against 14-3-3, occludin and claudin-5 proteins. In brief, the paraffin-embedded brain tissue sections (4-μm) were air-dried, deparaffinized with xylene, and rehydrated using gradient ethanol. After rinsing with PBS, the sections were heated in a microwave in sodium citrate buffer (0.01 mol/L, pH 6.0) for 20 minutes to retrieve antigens, and were then allowed to cool to room temperature. Endogenous peroxidase was quenched with 3% H2O2 for 10 minutes, and then the sections were blocked with blocking solution (10% goat serum in PBS with 0.3% Triton X-100) for 30 minutes. The sections were then incubated either with mouse monoclonal IgG for pan 14-3-3 and rabbit polyclonal IgG antibody for 14-3-3 β, ε, γ, η, ζ, τ, θ, σ, occludin and claudin-5 or with blocking solution without primary antibodies for 60 minutes at room temperature. A secondary biotinylated multilink antibody was then added for 30 minutes. After washing, streptavidin-horseradish peroxidase was applied for 20 minutes, followed by diaminobenzidine tetrahydrochloride in buffer for 10 minutes (Novocastra^TM^ Polymer Detection System; Leica Biosystems Newcastle Ltd., UK). The sections were then washed, dehydrated, and mounted.

### Statistical analysis

The concentrations of Evans blue, 14-3-3 protein isoforms and BBB junctional proteins occludin and claudin-5 in the different groups of mice were compared using the nonparametric Kruskal-Wallis test followed by post-testing using Dunn’s multiple comparison of means. The Mann Whitney U test was used to compare changes in 14-3-3 protein isoforms every week relative to the controls. All results were presented as medium and range. A p value less than 0.05 was considered to be statistically significant.

## Results

### BBB damage according to the Evans blue method and tight junctional proteins claudin-5 and occludin

**Fig 1:**
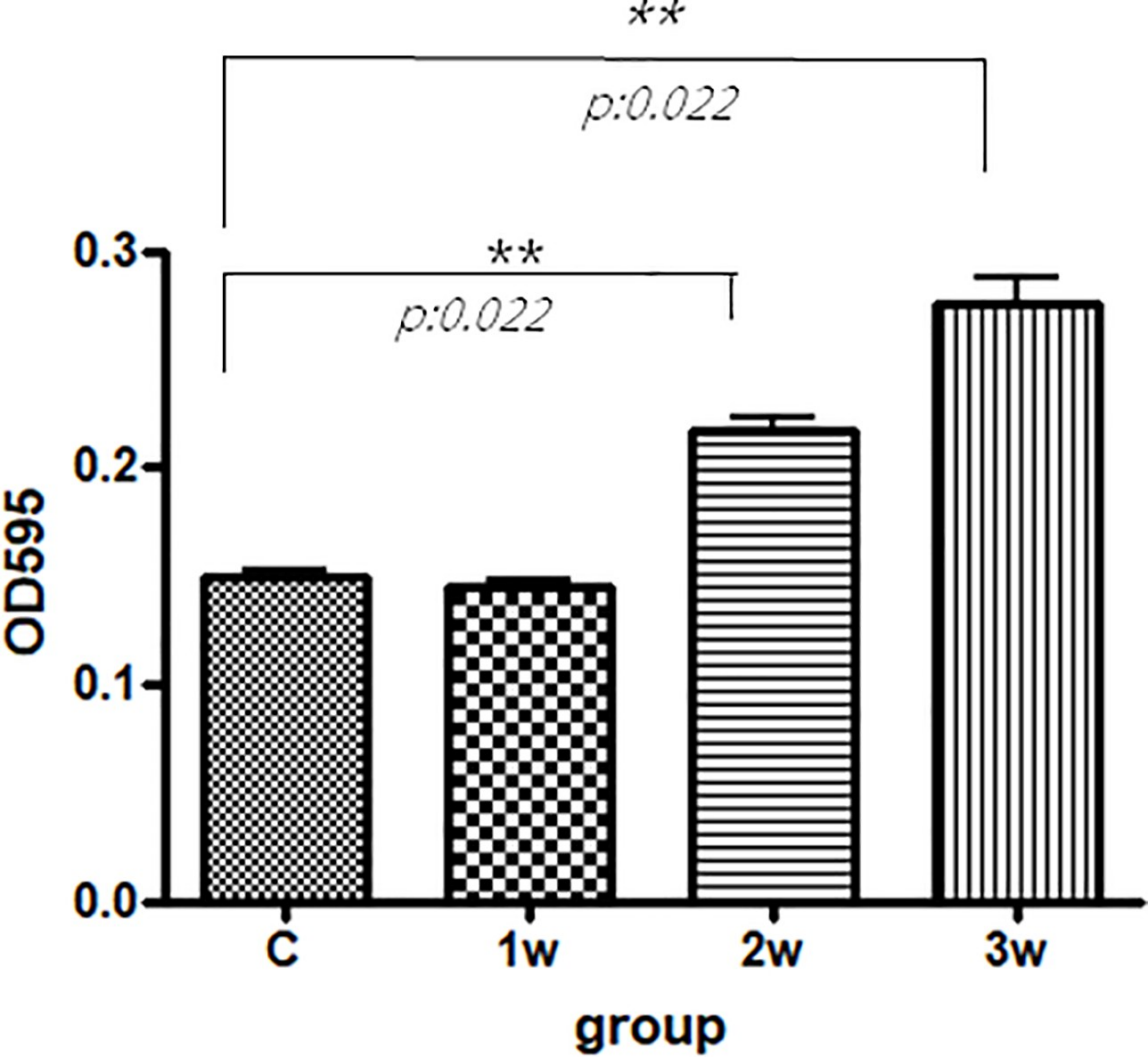
Dynamic changes in Evans blue concentrations in the brain homogenates of mice with *A*. *cantonensis* infection by intraperitoneal injection. The amount of Evans blue in the mice brain significantly increased 3 weeks after infection compared to the uninfected mice (p < 0.05). For each group, n = 5. All data are presented as mean ± SEM. Control: no parasitic infection; 1W: 1 week after infection; 2W: 2 weeks after infection; 3W: 3 weeks after infection.

**Fig 2:**
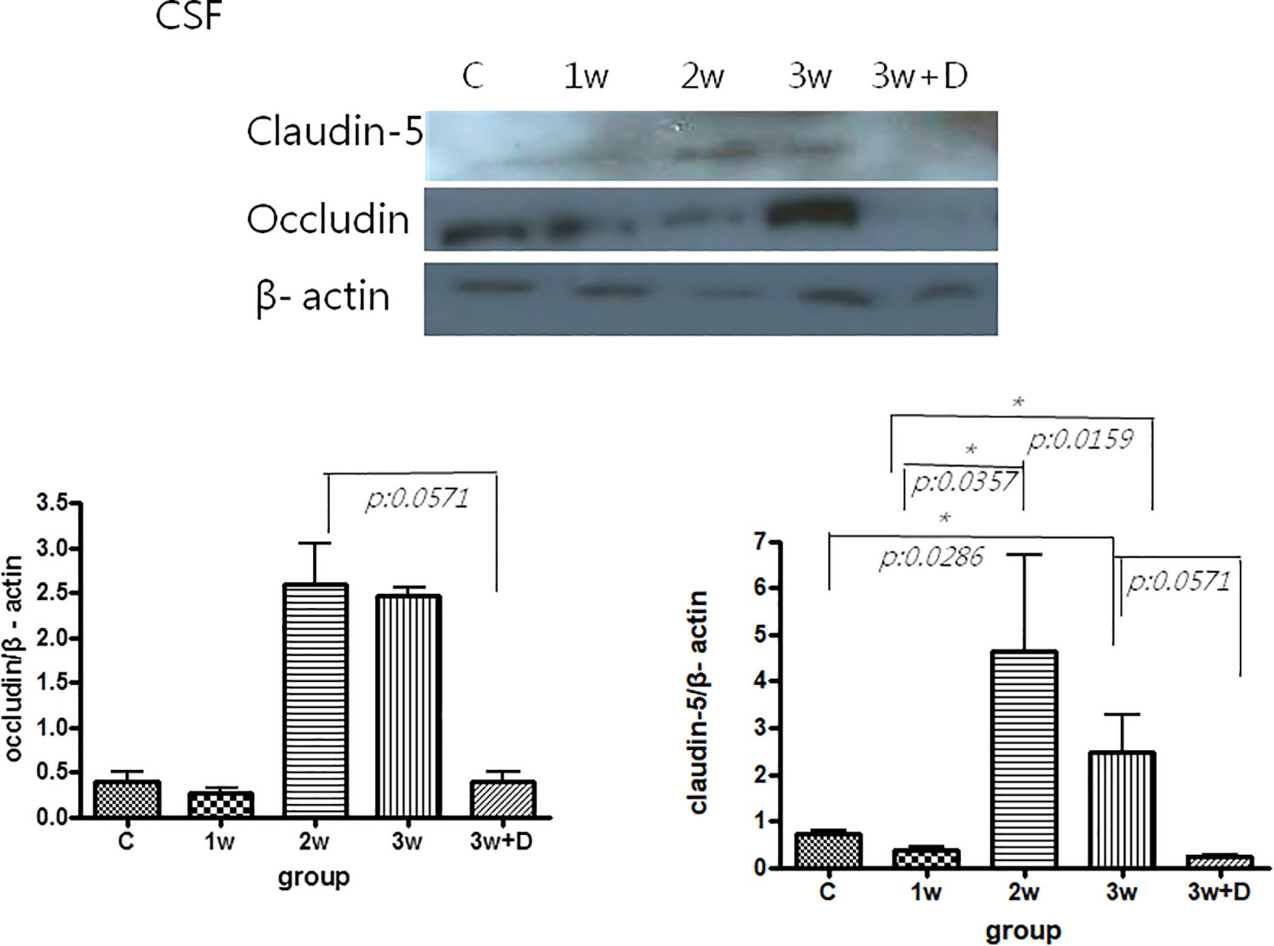
Western blot analysis of tight junctional proteins occludin and claudin-5 in the CSF of mice with *A*. *cantonensis* infection. The expressions of occludin and claudin-5 in the CSF tended to increase 3 weeks after infection compared to the uninfected mice (p = 0.057 and p = 0.0286, respectively). Dexamethasone administration only had marginal effect on the decrease of expressions of BBB junctional proteins occludin and claudin-5. Upper: Representative Western blots of claudin-5 and occludin in the different groups. Lower: Quantitation of claudin-5 and occludin in different groups. For each group, n = 5. All data are presented as mean ± SEM. Control: no parasitic infection; 1W: 1 week after infection; 2W: 2 weeks after infection; 3W: 3 weeks after infection; 3W + Dex: mice given dexamethasone for 2 weeks (7^th^ to 21^st^ day post infection) and sacrificed on day 21.

The concentrations of Evans blue in the infected mice brains were significantly increased 2 and 3 weeks after infection compared to the uninfected mice (p < 0.05). The expressions of CSF tight junctional proteins claudin-5 and occludin in Western blot analysis tended to be higher 3 weeks after infection compared to the uninfected mice (p = 0.0286 and p = 0.057, respectively). Therefore, BBB dysfunction was more severe in the mice 3 weeks after infection compared to the uninfected mice. Dexamethasone administration only partially decreased the expressions of BBB junctional proteins occludin and claudin-5 (Figs 1 and 2).

### Western blot analysis of 14-3-3 protein isoform concentrations in the CSF, brain homogenates and serum of the mice

**Fig 3:**
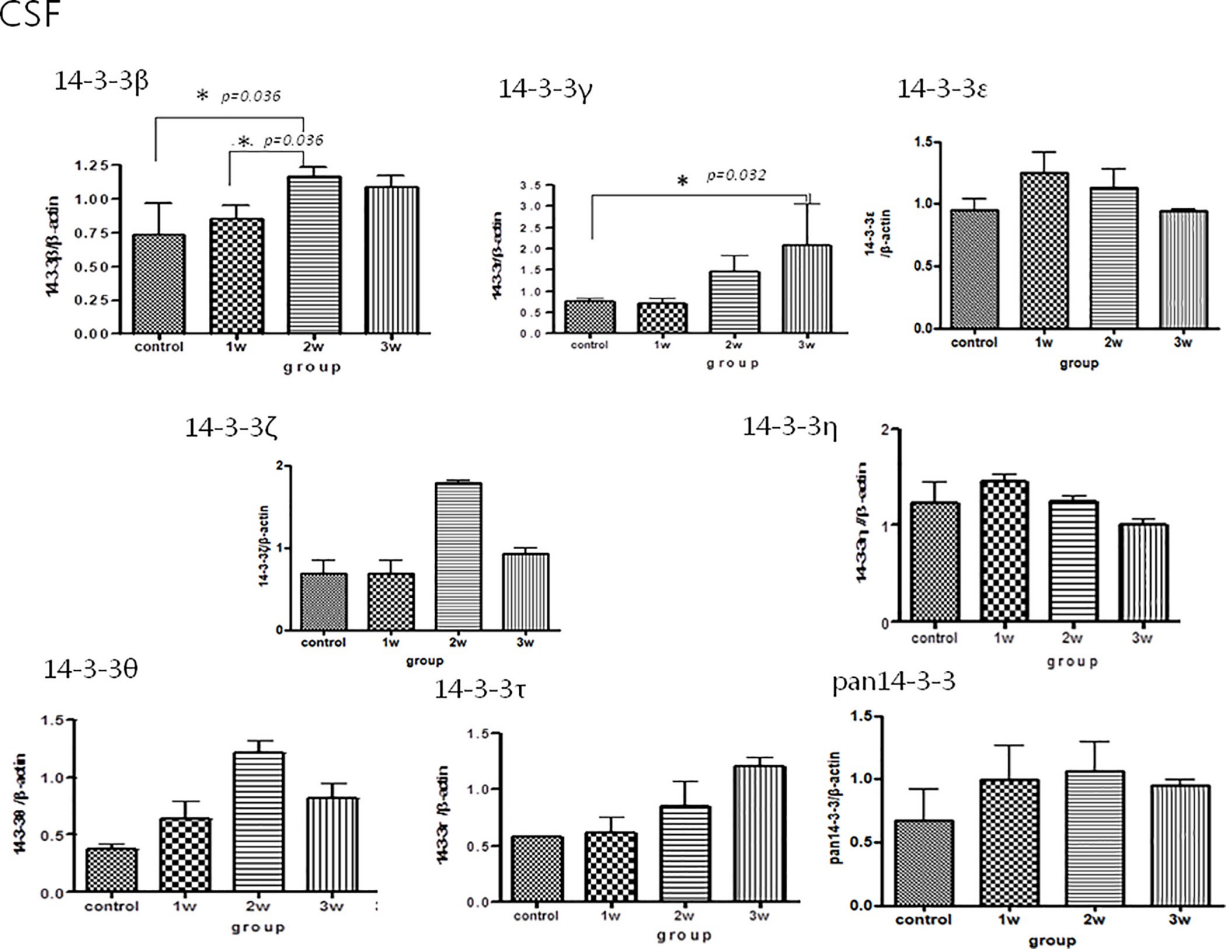
Western blot analysis of 14-3-3 protein isoform expressions in the CSF in the mice with *A*. *cantonensis* infection. There were significant increases in the amounts of 14-3-3 protein isoforms β and γ in the CSF in the third week after infection compared to the controls. Control: no parasitic infection; 1W: 1 week after infection; 2W: 2 weeks after infection; 3W: 3 weeks after infection.

**Fig 4:**
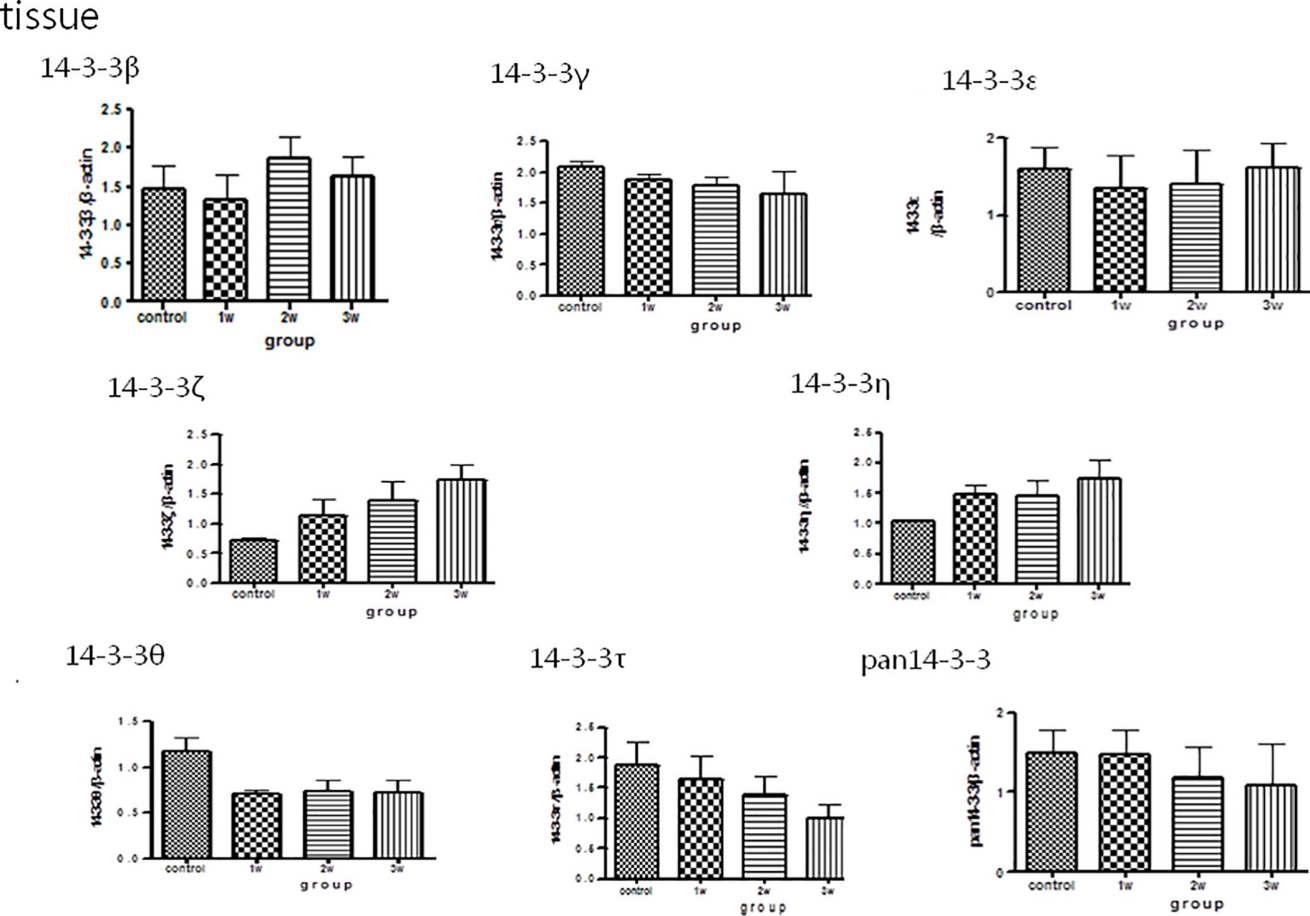
Western blot analysis of 14-3-3 protein isoform expressions in the brain homogenates in the mice with *A*. *cantonensis* infection. The expressions of 14-3-3 protein isoforms in the brain homogenates did not significantly change following 2–3 weeks of infection. Control: no parasitic infection; 1W: 1 week after infection; 2W: 2 weeks after infection; 3W: 3 weeks after infection.

**Fig 5:**
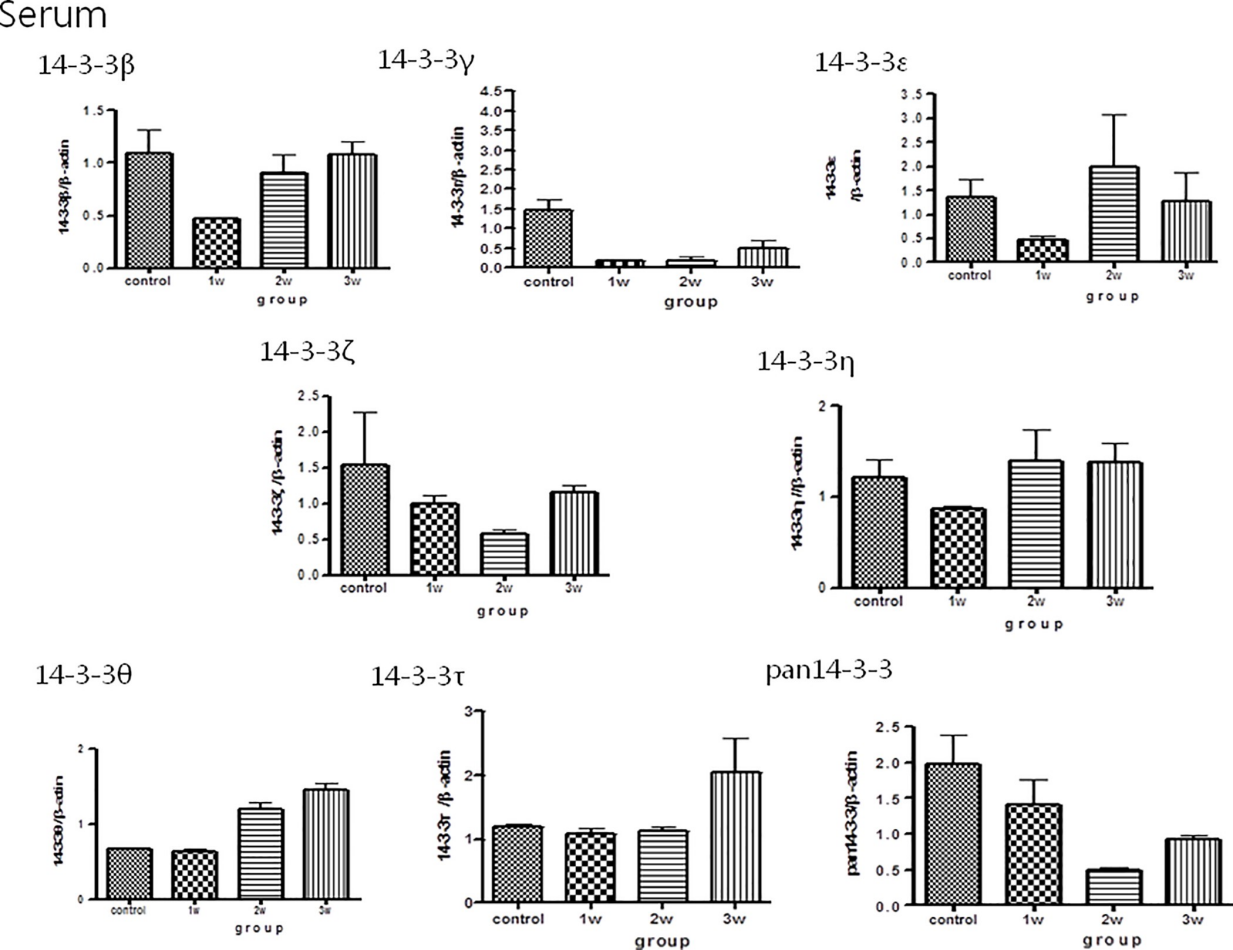
Western blot analysis of 14-3-3 protein isoform expressions in the serum in the mice with *A*. *cantonensis* infection. The expressions of 14-3-3 protein isoforms in the serum did not significantly change following 2–3 weeks of infection. Control: no parasitic infection; 1W: 1 week after infection; 2W: 2 weeks after infection; 3W: 3 weeks after infection.

The Western blot analysis for 14-3-3 protein isoforms in CSF/brain homogenates/serum is shown in Figs 3, 4 and 5. There were significant increases in 14-3-3 β and γ in the CSF in the third week after infection compared to the controls. There were no significant increases in other 14-3-3 isoform ε, η, ζ, τ, θ, and pan 14-3-3 following different weeks infection (Fig 3).The expressions of 14-3-3 protein isoforms in the brain homogenates and serum did not significantly change following 2–3 weeks of infection (Figs 4 and 5)

### Histopathological examination of mice brain tissue with *A*. *cantonensis* infection

**Fig 6:**
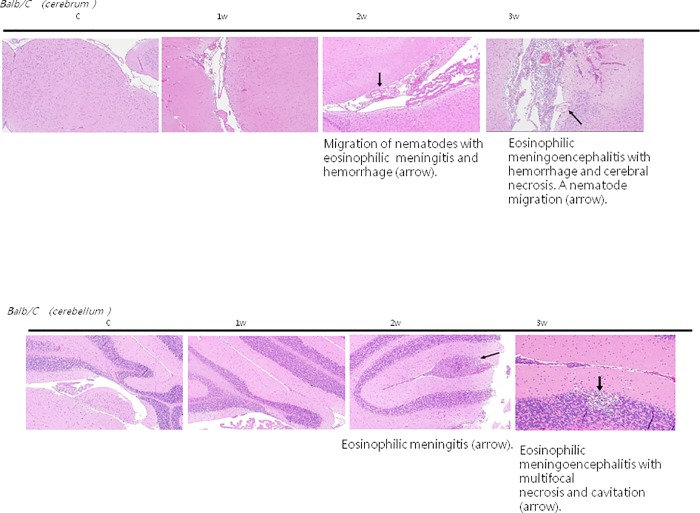
Mice brain hematoxylin and eosin staining. **It** revealed severe eosinophilic meningoencephalitis with hemorrhage, cerebral necrosis and nematode migration (arrow) in the cerebrum after 3 weeks of infection compared to the controls and those after 1–2 weeks of infection. Control: no parasitic infection; 1W: 1 week after infection; 2W: 2 weeks after infection; 3W: 3 weeks after infection.

Mice brain histology revealed **m**igration of nematodes with eosinophilic meningitis and hemorrhage (arrow) in the cerebrum after 2 weeks of infection and severe eosinophilic meningoencephalitis with hemorrhage, cerebral necrosis and nematode migration (arrow) in the cerebrum after 3 weeks of infection compared to the controls and those infected for 1 week. The multifocal necrosis and cavitations (Fig 6) in the cerebellum were also more severe after 3 weeks of infection compared to the controls and those infected for 1–2 weeks. This indicated that the BBB damage was more severe after 3 weeks of infection with *A*. *cantonensis*, as evidenced by increased Evans blue extravasation in the mice brain homogenates after 3 weeks of infection.

### IHC staining of 14-3-3 protein isoforms and BBB junctional proteins occludin and claudin-5 in the meninges of the mice brains

**Fig 7:**
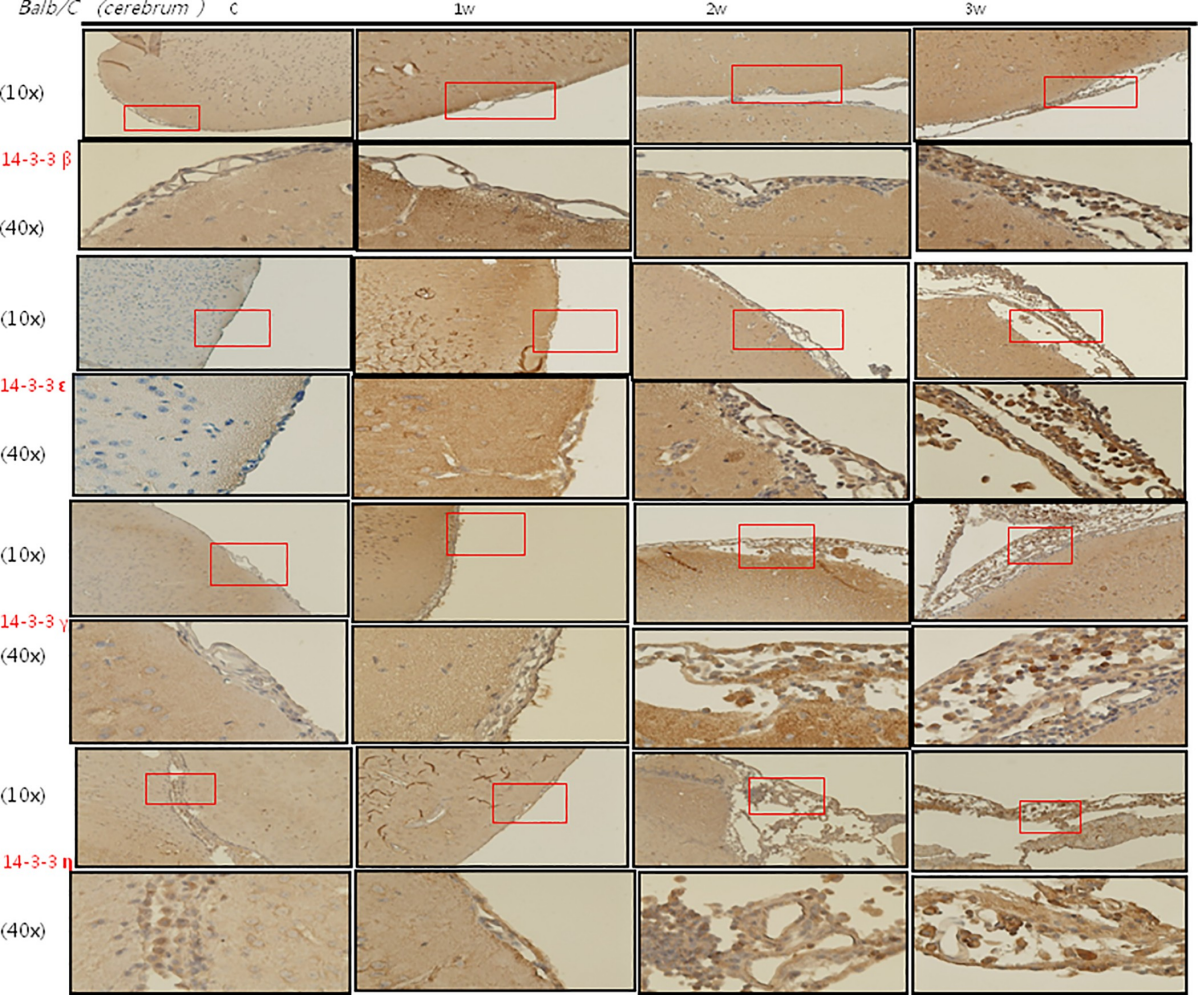
IHC studies for 14-3-3 protein isoforms β, ε, γ and η in the meninges of the cerebrum. IHC studies showing significant increases in 14-3-3 protein isoforms β, γ and ε in the cerebrum meninges in the second and third weeks after infection compared to the controls and those infected for 1 week. Scale bar, 10 um at 10x and 40x magnification. Control: no parasitic infection; 1W: 1 week after infection; 2W: 2 weeks after infection; 3W: 3 weeks after infection.

**Fig 8:**
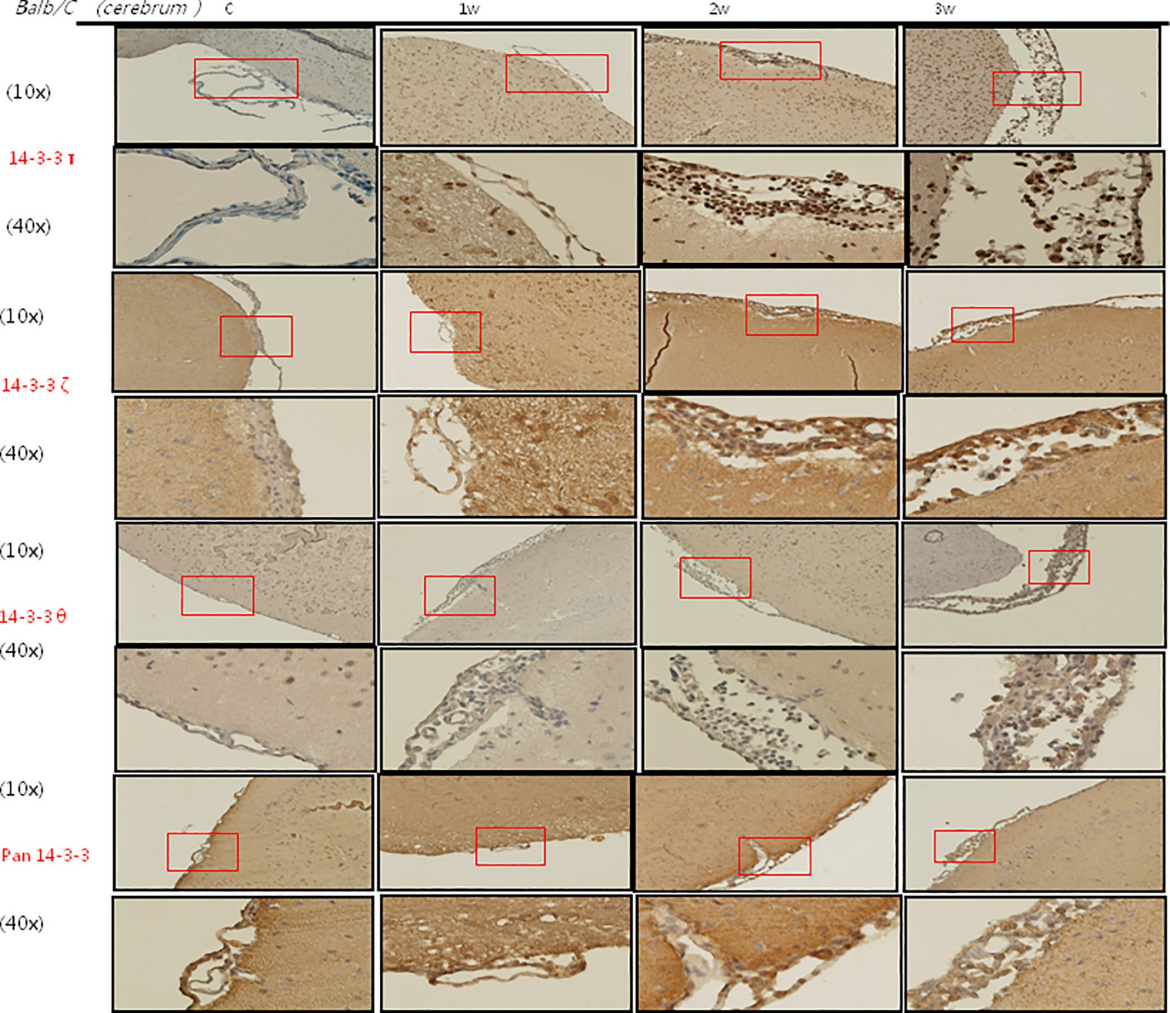
IHC studies for 14-3-3 protein isoforms τ, ζ, θ and pan 14-3-3 in the meninges of the cerebrum. IHC studies showing significant increases in 14-3-3 protein isoforms θ in the cerebrum meninges in the second and third weeks after infection compared to the controls and those infected for 1 week. Scale bar, 10 um at 10x and 40x magnification. Control: no parasitic infection; 1W: 1 week after infection; 2W: 2 weeks after infection; 3W: 3 weeks after infection.

**Fig 9:**
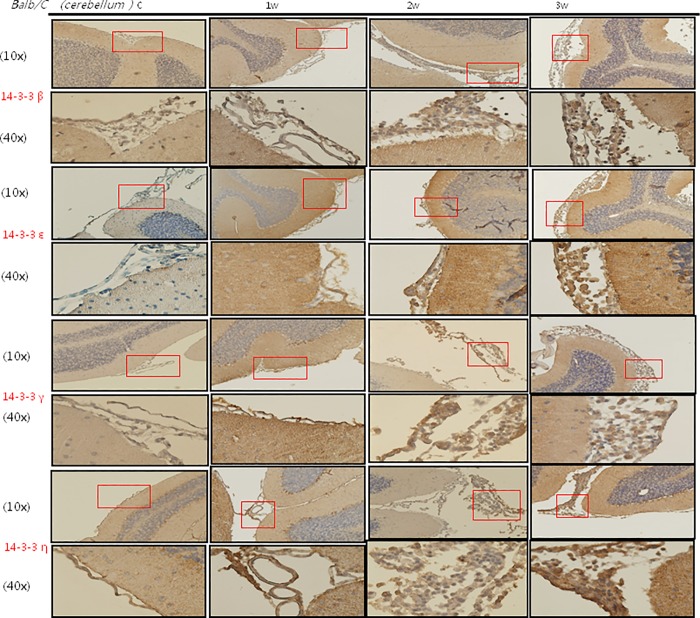
IHC studies for 14-3-3 protein isoforms β, ε, γ and η in the meninges of the cerebellum. IHC studies showing significant increases in 14-3-3 protein isoforms β, γ and ε in the cerebellum in the second and third weeks after infection compared to the controls and those infected for 1 week. Scale bar, 10 um at 10x and 40x magnification. Control: no parasitic infection; 1W: 1 week after infection; 2W: 2 weeks after infection; 3W: 3 weeks after infection.

**Fig 10:**
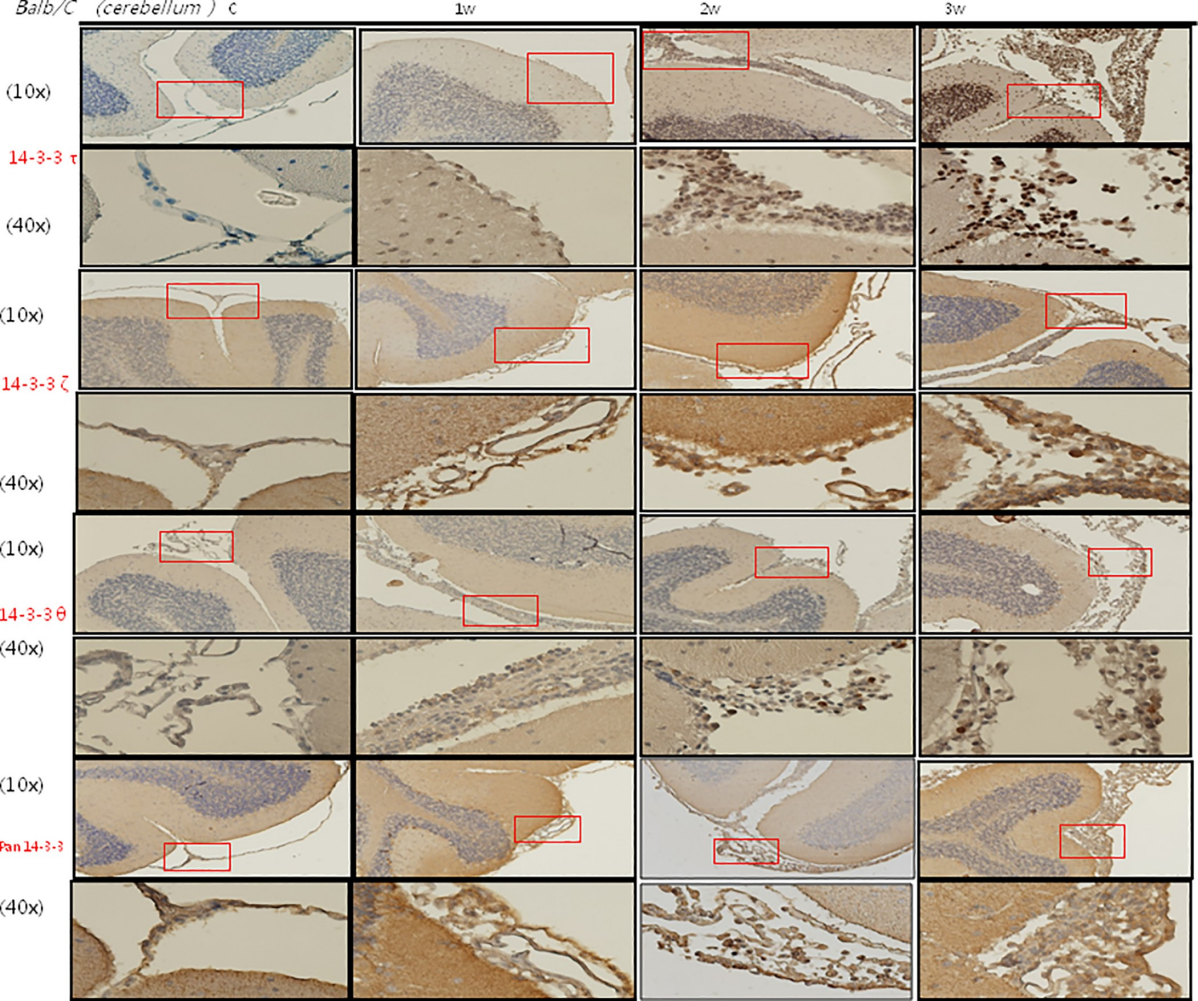
IHC studies for 14-3-3 protein isoforms τ, ζ, θ and pan 14-3-3 in the meninges of the cerebellum. IHC studies showing significant increases in 14-3-3 protein isoforms θ in the cerebellum meninges in the second and third weeks after infection compared to the controls and those infected for 1 week. Scale bar, 10 um at 10x and 40x magnification. Control: no parasitic infection; 1W: 1 week after infection; 2W: 2 weeks after infection; 3W: 3 weeks after infection.

**Fig 11:**
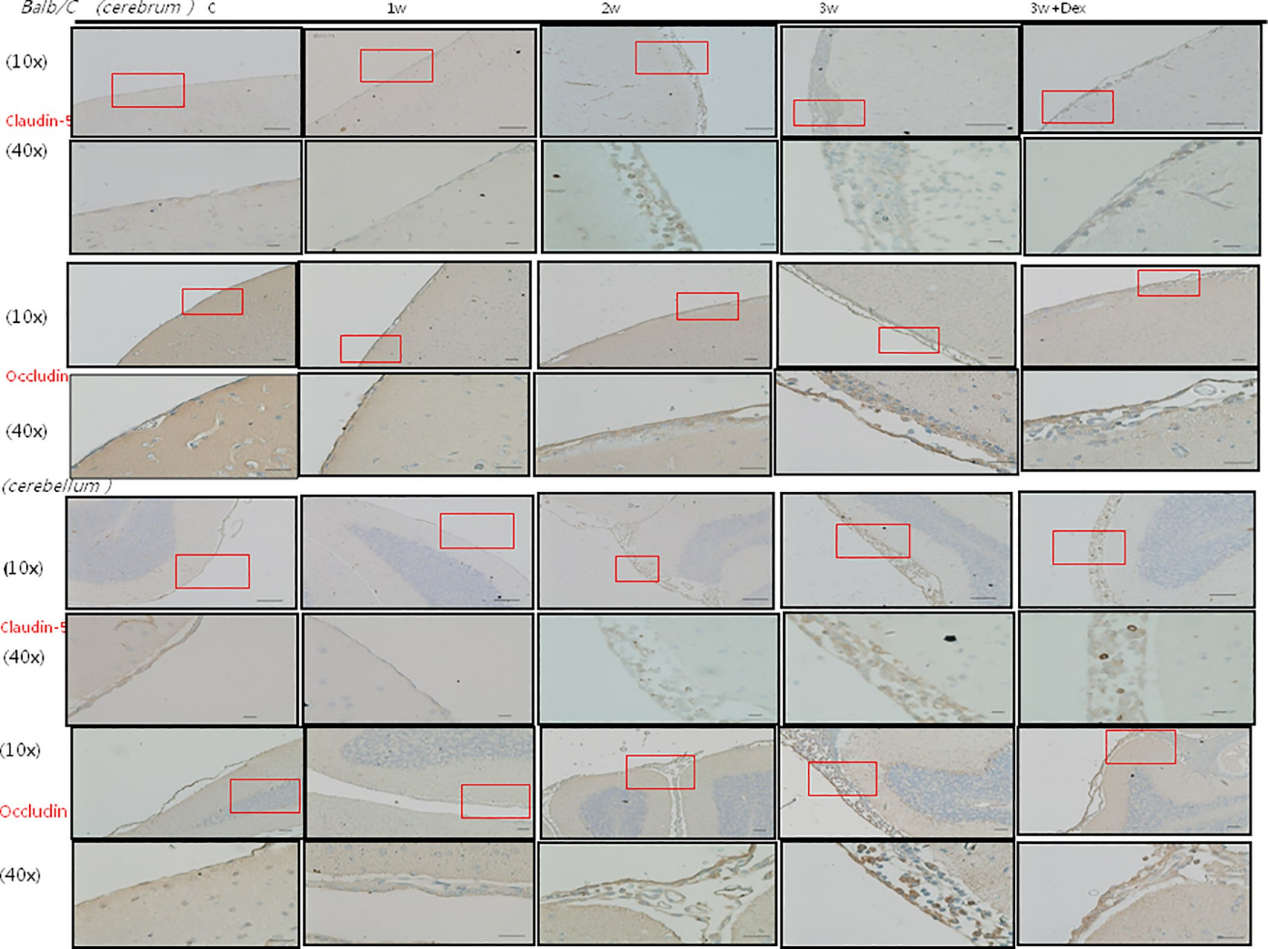
The IHC studies for BBB junctional proteins occludin and claudin-5. IHC studies showing significant increases in occludin and claudin-5 expressions in the brain meninges in the second and third weeks after infection compared to the controls and those infected for 1 week. Dexamethasone administration significantly decreased the expressions of occludin and claudin-5 in the mice meninges. Scale bar, 10 um at 10x and 40x magnification. Control: no parasitic infection; 1W: 1 week after infection; 2W: 2 weeks after infection; 3W: 3 weeks after infection; 3W + Dex: mice given dexamethasone for 2 weeks (7^th^ to 21^st^ day post infection) and sacrificed on day 21.

The IHC studies for 14-3-3 protein isoforms in the meninges of the cerebrum and cerebellum are shown in Figs 7, 8, 9 and 10 IHC staining was mainly seen in meningeal inflammatory cells. There were significant increases in 14-3-3 β, γ, ε and θ in the meninges of the cerebrum and cerebellum in the second and third weeks after infection compared to the controls and those infected for 1 week. However, there were no significant changes in 14-3-3 η, ζ and τ isoforms or pan 14-3-3 in the meninges of the cerebrum and cerebellum in the second and third weeks after infection compared to the controls and those infected for 1 week. The IHC studies for BBB junctional proteins occludin and claudin-5 are shown in Fig 11. There were significant increases in occludin and claudin-5 expressions in the meninges of the cerebrum and cerebellum in the second and third weeks after infection compared to the controls and those infected for 1 week. Dexamethasone administration significantly decreased the expressions of junctional proteins occludin and claudin-5 in the mice meninges. This experiment further demonstrated that BBB dysfunction was most severe 2–3 weeks after infection compared to the controls and 1 week after infection.

## Discussion

In this study, we found significant increases in CSF 14-3-3 protein isoforms β and γ in the second and third weeks after infection compared to the controls and those infected for 1 week, which was consistent with the severity of BBB damage as assessed using the Evans blue assay, hematoxylin and eosin staining and increased expressions of tight junctional proteins occludin and claudin-5 in Western blot analysis and IHC studies. Using IHC to assess the distribution of 14-3-3 protein isoforms in the mice brain meninges, we also found that the expressions of 14-3-3 protein isoforms β, γ, ε and τ/θ in the brain meninges increased over a 3-week period after infection.

An animal model of parasitic eosinophilic meningitis caused by *A*. *cantonensis* conducted several decades ago showed that infective larvae migrate from the gastrointestinal tract to the CNS by hematogenous spread [25]. Approximately 10 days after infection, the larvae appeared in the subarachnoid space then gradually spread to the cerebral hemispheres, and they remained in the subarachnoid space from 8 days to several weeks, provoking a severe acute inflammatory, granulomatous reaction and direct or mechanical damage [25]. The time course of brain destruction is consistent with the present study, in that the most severe BBB damage occurred in the second and third weeks of infection with simultaneous increases in the expressions of 14-3-3 protein isoforms β and γ in the CSF.

The 14-3-3 proteins belong to a family of acidic, dimeric proteins that are expressed in all eukaryotic cells. The detailed functions of these proteins were described by Berg et al. [26], and include regulating apoptosis, cell differentiation and senescence, coordination of cell adhesion and motility, maintenance of cell cycle checkpoints and DNA repair [26]. The highest tissue concentration of 14-3-3 proteins is in the brain, comprising about 1% of its total soluble protein [27]. In addition to their possible role in neuronal function, 14-3-3 proteins have attracted increasing interest owing to their possible involvement in the pathophysiology of various neurological disorders [12, 20, 23, 28, 29]. Different 14-3-3 isoforms have been implicated in the regulation of many intracellular signaling processes via their ability to bind specific phospho-serine/threonine-containing motifs on the target protein [26]. Furthermore, the 14-3-3 proteins have been shown to regulate the mitogen-activated protein kinase (MAPK) pathway [30]. Seven 14-3-3 isoforms have been identified: β, ε, γ, η, ζ, τ/θ and σ. As described in a previous study [30], the 14-3-3 θ isoform is bound to Bax in the cytoplasm, which undergoes dissociation from 14-3-3 θ during apoptosis via caspase-independent and -dependent mechanisms, to induce apoptotic changes of the mitochondria. In a mice brain astrocyte study, Chen et al. showed that the sonic hedgehog (Shh) signaling pathway may reduce cell apoptosis by inhibiting oxidative stress in *A*. *cantonensis* infection. They also demonstrated that excretory/secretory products of fifth-stage larvae of *A*. *cantonensis* could stimulate astrocyte activation and cytokines IL-1β and IL-6 through NF-κB and the Shh signaling pathway [31, 32]. Further studies are needed to investigate interactions between the 14-3-3 proteins and the Shh pathway.

Several studies have demonstrated that the 14-3-3 β isoform is a neuropathological marker which can be used to monitor neuronal damage in patients with bacterial and eosinophilic meningitis [12, 23]. Increased levels of 14-3-3 and five isoforms (β, γ, ε, η and ζ) were also reported in the CSF of 29 adults with community-acquired bacterial meningitis [17]. In addition, elevated levels of 14-3-3 γ in the CSF of HIV-negative patients with cryptococcus meningitis have been reported, with no change in the level after short-term treatment [18].

In a Japanese study, researchers found three isoforms (γ, ε and ζ) in the CSF of patients with HIV and AIDS dementia complex or cytomegalovirus encephalitis, but not in patients with AIDS without neurological symptoms or in those without HIV [33]. Moreover, Morassutti et al. reported that the 14-3-3 proteins reacted with 31-kDa antigen in *A*. *cantonensis* infection [34]. In the present study, we showed that the dynamic changes in the expressions of 14-3-3 protein isoforms β and γ in the CSF and brain meninges in our mice model were correlated with the dynamic changes in BBB dysfunction as demonstrated by the Evans blue assay, hematoxylin and eosin staining, and changes in the expressions of the tight junctional proteins occludin and claudin-5. Furthermore, not all of the 14-3-3 protein isoforms in the CSF/brain meninges had similar increases after 2–3 weeks of infection, which is in agreement with the previous findings that 14-3-3 γ is brain specific and 14-3-3 β is a neuropathological marker. Further studies are needed to clarify these findings.

Our results suggest that the 14-3-3 proteins could be a marker of neuronal damage, and that the presence of 14-3-3 protein isoforms in the CSF may be the consequence of disruption of the BBB caused by eosinophilic meningitis, as it has been shown that infection by a parasite can induce the up-regulation of MMP-9 [35], vascular endothelial growth factor [36] and hepatocyte growth factor [37], and contribute to the degradation of meningeal blood vessel membranes. This change in the cerebral vasculature could account for leakage of 14-3-3, primarily a CNS protein, into the CSF circulation.

Further studies are needed to elucidate why some of the 14-3-3 protein isoforms in the mice brain homogenates were not significantly increased after infection. It may be due to the uneven distribution of 14-3-3 protein isoforms in mice brains and the mechanical injury caused by parasite invasion. It may also be due to the semi-quantitative nature of Western blot analysis, which may help to explain the small increase in serum 14-3-3 protein isoforms after infection.

There are several limitations to this study. First, we used the less sensitive Evans blue method to monitor BBB dysfunction. However, the dynamic changes in brain tissue according to hematoxylin and eosin staining and the expressions of occludin and claudin-5 in the CSF and brain meninges were also consistent with the severity of BBB damage. Quantifying the level of albumin in serum and CSF and calculating the CSF/serum albumin ratio would have improved the sensitivity. Second, the animal samples were analyzed using semi-quantitative Western blot analysis, and this would decrease the sensitivity and specificity. Furthermore, the sources of 14-3-3 proteins needed to be clarified. Given the presence of 14-3-3 proteins in the uninfected control mice, it is possibly that the 14-3-3 proteins came from the mice. However, McGowan et al. [38] reported that the *A*. *cantonensis* genome has 80% sequence identity to the human 14-3-3 z isoform. It is possible that the polyclonal antibody of 14-3-3 proteins could have reacted with 14-3-3 proteins from *A*. *cantonensis*. The increased parasitic load 3 weeks after infection may have led to increases in 14-3-3 protein expressions in the CSF and meninges, as well as the release of mice brain 14-3-3 proteins caused by the mechanical injury of larvae invasion to the CNS. Finally, the data obtained in the context of the mice model may not represent the pathogenesis of human infection.

## Conclusions

The results of the current study suggest that the mice with eosinophilic meningitis with increased expressions of 14-3-3 protein isoforms β and γ in the CSF and brain meninges tended to have more severe dysfunction of the BBB as evidenced by Evans blue staining, brain hematoxylin and eosin staining, and changes in the expressions of the tight junctional proteins occludin and claudin-5. These results shed light on the role of 14-3-3 proteins in eosinophilic meningitis caused by *A*. *cantonensis*.

## Supporting information

S1 Fig BBB junctional proteins occludin and claudin-5 in brain homogenates. (TIF)

S1 File NC3Rs ARRIVE guidelines checklist. (PDF)

S2 File ATS editing certificate. (PDF)

S3 File Dynamic changes of Evans blue extravasation. (XLSX)

S4 File Dynamic changes of 14-3-3 proteins in CSF. (XLSX)

S5 File Dynamic changes of 14-3-3 proteins in serum. (XLSX)

S6 File Dynamic changes of 14-3-3 proteins in brain tissues. (XLSX)

S7 File Dynamic changes of occludin and claudin-5 in CSF. (XLS)

S8 File Dynamic changes of occludin and claudin-5 in brain tissues. (XLS)
