# Supplementary figures and images for: Increased 14-3-3β and γ protein isoform expressions in parasitic eosinophilic meningitis caused by Angiostrongylus cantonensis infection in mice
Source: PLoS One. 2019 Mar 7;14(3):e0213244. doi: 10.1371/journal.pone.0213244 (PMC6405114; doi:10.1371/journal.pone.0213244)

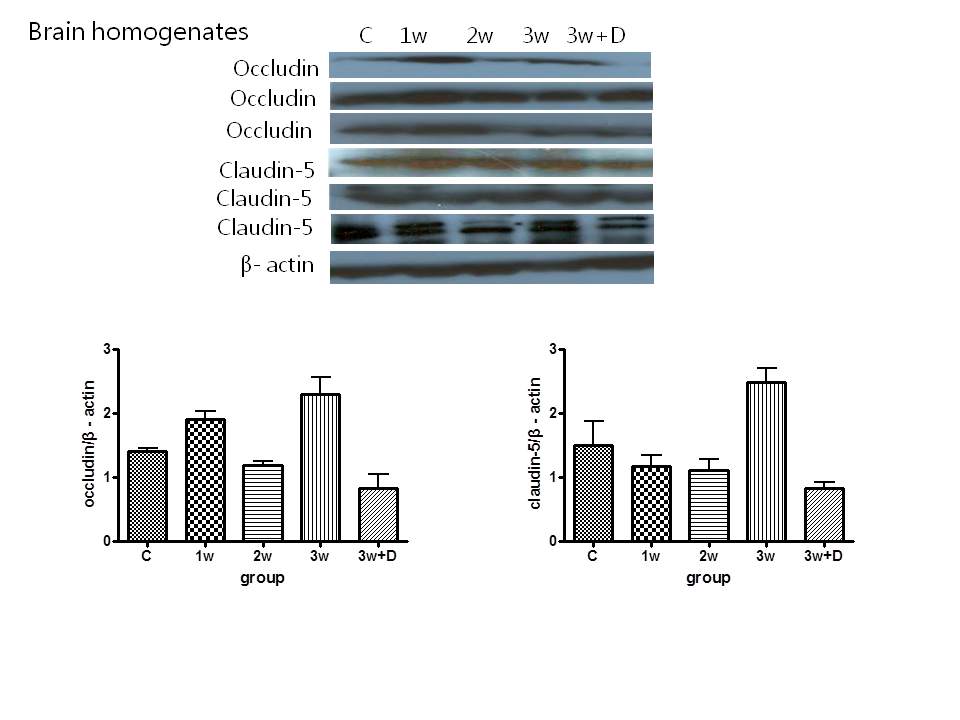

Supplement: S1 Fig — (TIF) [file pone.0213244.s001.tif]
