# Supplementary material for: Increased 14-3-3β and γ protein isoform expressions in parasitic eosinophilic meningitis caused by Angiostrongylus cantonensis infection in mice
Source: PLoS One. 2019 Mar 7;14(3):e0213244. doi: 10.1371/journal.pone.0213244 (PMC6405114; doi:10.1371/journal.pone.0213244)
Supplement: S2 File — (PDF) [file pone.0213244.s003.pdf]

ATS
Medical Editing
亞洲通顧問有限公司

Medical Editing Certificate

This is to certify that the following manuscript has been edited by an experienced native English speaking medical editor.

Title:

Increased 14-3-3β and γ Protein Isoform Expressions in Parasitic Eosinophilic Meningitis Caused by *Angiostrongylus cantonensis* Infection in Mice

Authors:

Hung-Chin Tsai, Yu-Hsin Chen, Chuan-Min Yen, Susan Shin-Jung Lee, and Yao-Shen Chen

Date:

February 13, 2019

Please note that although the manuscript was edited for English language, grammar, punctuation, spelling, and readability on the date listed above, the author(s) ultimately made the decision to accept or reject our changes and suggestions.
